# Gli Regulates *MUC5AC* Transcription in Human Gastrointestinal Cells

**DOI:** 10.1371/journal.pone.0106106

**Published:** 2014-08-28

**Authors:** Natsuko Kageyama-Yahara, Nobutake Yamamichi, Yu Takahashi, Chiemi Nakayama, Kazuya Shiogama, Ken-ichi Inada, Maki Konno-Shimizu, Shinya Kodashima, Mitsuhiro Fujishiro, Yutaka Tsutsumi, Masao Ichinose, Kazuhiko Koike

**Affiliations:** 1 Department of Gastroenterology, Graduate School of Medicine, The University of Tokyo, Bunkyo-ku, Tokyo, Japan; 2 1st Department of Pathology, Fujita Health University School of Medicine, Toyoake, Aichi, Japan; 3 Second Department of Internal Medicine, Wakayama Medical College, Kimiidera, Wakayama, Japan; Wayne State University, United States of America

## Abstract

MUC5AC is a well-known gastric differentiation marker, which has been frequently used for the classification of stomach cancer. Immunohistochemistry revealed that expression of MUC5AC decreases accompanied with increased malignant property of gastric mucosa, which further suggests the importance of *MUC5AC* gene regulation. Alignment of the 5′-flanking regions of *MUC5AC* gene of 13 mammal species denoted high homology within 200 bp upstream of the coding region. Luciferase activities of the deletion constructs containing upstream 451 bp or shorter fragments demonstrated that 15 bp region between −111 and −125 bp plays a critical role on *MUC5AC* promoter activity in gastrointestinal cells. We found a putative Gli-binding site in this 15 bp sequence, and named this region a highly conserved region containing a Gli-binding site (HCR-Gli). Overexpression of Gli homologs (Gli1, Gli2, and Gli3) clearly enhanced *MUC5AC* promoter activity. Exogenous modulation of Gli1 and Gli2 also affected the endogenous *MUC5AC* gene expression in gastrointestinal cells. Chromatin immunoprecipitation assays demonstrated that Gli1 directly binds to HCR-Gli: Gli regulates *MUC5AC* transcription via direct protein-DNA interaction. Conversely, in the 30 human cancer cell lines and various normal tissues, expression patterns of MUC5AC and Gli did not coincide wholly: MUC5AC showed cell line-specific or tissue-specific expression whereas Gli mostly revealed ubiquitous expression. Luciferase promoter assays suggested that the far distal *MUC5AC* promoter region containing upstream 4010 bp seems to have several enhancer elements for gene transcription. In addition, treatments with DNA demethylation reagent and/or histone deacetylase inhibitor induced *MUC5AC* expression in several cell lines that were deficient in MUC5AC expression. These results indicated that Gli is necessary but not sufficient for *MUC5AC* expression: namely, the multiple regulatory mechanisms should work in the distal promoter region of *MUC5AC* gene.

## Introduction

Although the mortality and incidence of gastric cancer has gradually fallen in the last several decades, it is still the fourth most common malignancy and the second leading cause of cancer-related death worldwide [1]. Gastric cancer mainly occurs from gastric mucosa with intestinal metaplasia, which is mostly caused by chronic infection of *Helicobacter pylori* [2], [3]. Intestinal metaplasia in stomach is typically classified into two types: mixed gastric-and-intestinal type (incomplete type) and solely intestinal type (complete type) [4]. In the process of metaplastic change of gastric mucosa, induction of intestinal differentiation and loss of gastric differentiation occur simultaneously or asynchronously [5], [6].

For induction of intestinal differentiation, we and other groups have been reported that Cdx must be an indispensable key molecule by regulating transcription of many intestinal marker genes [7], [8], [9], [10], [11], [12], [13]. On the contrary, the molecular mechanism of loss of gastric genes in the process of metaplastic change in stomach remains poorly understood. It is probably due to inadequate identification of gastric marker genes, and also due to unsolved expression regulation of these gastric genes. Based on these backgrounds, we focused on *MUC5AC*, which is a well-established gastric marker gene [6], [14] and is often used for the clinical assessment of gastric cancer [15]. There have been many previous reports investigating the expression of *MUC5AC* and prognosis of gastric cancer, but the association between expression of *MUC5AC* and malignant potential of gastric cancer is still controversial [16], [17], [18], [19]. Nevertheless, *MUC5AC* is one the most evident gastric marker clearly decreased in the process of intestinal metaplasia [14], [20]. We believe elucidating the mechanism of *MUC5AC* gene expression in gastrointestinal cells must be useful to understand the loss of gastric differentiation during the development of pre-malignant atrophic gastritis.

Our aim of this study is to find a critical mechanism of *MUC5AC* expression regulation in human gastrointestinal cells. To date, HIF-1α [21], Smad4 [21], [22], Sp1 [22], GATA-4/-6, and HNF-1/-4 [23] are reported to activate murine *MUC5AC*. Sp1 and Gli are reported to enhance *MUC5AC* expression in human lung-epithelial and pancreatic cancer cells, respectively [24], [25]. Contrastively, ATBF1 is negatively regulate *MUC5AC* expression in human gastric cancer cells [26], In spite of these results, molecular mechanisms of MUC5AC expression in human gastrointestinal cells have not been fully elucidated. In the present study, we identified 15 bp sequence in human *MUC5AC* 5′-flanking region which plays an important role in *MUC5AC* expression in gastrointestinal cancer cells, and also found that the region contains a putative Gli-binding site which does not coincide with previous report [25]. Gli is one of transcriptional factors which has a DNA binding zinc finger domain [27]. In this study, we examined molecular roles of Gli on *MUC5AC* promoter in gastrointestinal cells. Our results demonstrated that *MUC5AC* expression depends on cooperative regulatory mechanism of Gli, some epigenetic modulation, and other factors in gastrointestinal cells.

## Materials and Methods

### Cell Culture

Twenty gastric cancer cell lines, ten colorectal cancer cell lines, and two non-gastrointestinal cancer cell lines were maintained in high-glucose DMEM with 10% fetal calf serum (Gibco/Invitrogen, Carlsbad, CA) at 37°C in a humidified 5% CO_2_ atmosphere. Names of used cell lines and histological types of gastric cancer cell lines were described in our previous reports [6]. Human T98G and A172 cell lines were purchased from the RIKEN Bio Resource Center (Tsukuba, Japan). For the treatment with DNA demethylation reagent or histone deacetylase (HDAC) inhibitor, 5-Aza-2′-deoxycytidine (5-Aza-dC, Sigma-Aldrich) at 2 µg/ml and/or trichostatin A (TSA, Sigma-Aldrich) at 25–1000 ng/ml were added to the culture medium.

### Tumor Samples

For the advanced gastric cancer specimens, we randomly selected 89 gastric adenocarcinoma samples surgically resected at the Fujita Health University Hospital. For the early stage gastric cancer endoscopically resected, we selected 78 specimens banked at the University of Tokyo Hospital. This study was approved by the ethic committees of the University of Tokyo, and also by the institutional ethical review board for human investigation at Fujita Health University. According to the Declaration of Helsinki, written informed consents were obtained from all the study participants for use of resected sample in research.

### Immunohistochemistry

Deparaffinization and endogenous peroxidase inactivation of clinical tissues were performed as described previously [28]. For *MUC5AC*, hydrated heating in 1 mM EDTA buffer (pH 8.0) at 120°C was then performed in a pressure cooker (Delicio 6L; T-FAL, Rumily, France) for 10 min for antigen retrieval. The primary immunostaining with anti-MUC5AC antibody (NCL-MUC-5AC, Novocastra, Newcastle-upon-Tyne, UK) at a 1∶200 dilution was applied for 12 hours at room temperature. After washing in PBS three times, the secondary immunostaining with Histofine Simple Stain MAX-PO(G) (Nichirei, Tokyo, Japan) was applied for 30 min at room temperature. Based on the evaluation by the two independent pathologists, ratios of *MUC5AC*-positive cells were classified into four classes: 1) <10%, 2) ≥10% and <50%, 3) ≥50% and <80%, and 4) ≥80%.

For Gli1 immunostaining, hydrated heating in 10 mM citrate buffer (pH 6.0) was done in a pressure cooker for 10 min for antigen retrieval. The sections were then incubated for 1 h at room temperature with antihuman Gli1 antibody (H-300, sc-20867, Santa Cruz Biotechnology) at a 1∶500 dilution. For the amplification of signals, anti-rabbit antibody (Dako, Hamburg, Germany) was applied to the slides for 30 min at room temperature. This was followed by incubation with FITC-conjugated phenol (fluorescyl-tyramide; Dako) for 15 min at room temperature and incubation with anti-FITC antibody conjugated to HRP (Dako) for 15 min at room temperature was then done.

### Plasmid constructions

The primer sequences used in plasmid constructions are listed in Table 1. To construct the vectors for luciferase reporter assays, the 2000 bp and 1433 bp of *MUC5AC* promoter region were amplified from TIG-112 genome using the primers MUC5ACup-F-01/MUC5ACup-R-01 and MUC5ACup-F-02/MUC5ACup-R-01. The amplified products were cloned into pT7blue-T-vector (Novagen, Darmstadt, Germany), and then digested with SalI and BamHI. The excised 2 kb and 1.4 kb DNA fragments were inserted into the XhoI/BglII site of pGL4.12 (Promega, Madison, WI, USA) to generate pGL4.12-hMUC5ACup2000bp and pGL4.12-hMUC5ACup1433bp. NcoI-HindIII fragment from pGL4.12-hMUC5ACup2000bp was inserted into the EcoRV/HindIII site of pGL4.12 to generate pGL4.12-hMUC5ACup451bp. The 280 bp, 190 bp, 158 bp, 150 bp, 138 bp, 125 bp and 110 bp of *MUC5AC* promoter region were amplified from pGL4.12-hMUC5ACup451bp as a template using primers MUC5ACup-280-XhoI, MUC5ACup-190-XhoI, MUC5ACup-158-XhoI, MUC5ACup-150-XhoI, MUC5ACup-138-XhoI, MUC5ACup-125-XhoI, MUC5ACup-110-XhoI and pGL412-HindIII, digested with HindIII and XhoI, and inserted into the HindIII/XhoI site of pGL4.12 to generate pGL4.12-hMUC5ACup280bp, pGL4.12-hMUC5ACup190bp, pGL4.12-hMUC5ACup158bp, pGL4.12-hMUC5ACup150bp, pGL4.12-hMUC5ACup138bp, pGL4.12-hMUC5ACup125bp and pGL4.12-hMUC5ACup110bp. The 4010 bp to 1432 bp upstream of MUC5AC promoter region was amplified from genome of TIG-112 genome using the primers MUC5ACup-SacI-F and MUC5ACup-PciI-R, digested with SacI and PciI, and inserted into the SacI/PciI site of pGL4.12-hMUC5ACup2000bp to generate pGL4.12-hMUC5ACup4010bp. The 3000 bp to 1432 bp upstream of MUC5AC promoter region was amplified from TIG-112 genome using the primers MUC5ACup-3000-NheI-F and MUC5ACup-PciI-R, digested with NheI and PciI, and inserted into the NheI/PciI site of pGL4.12-hMUC5ACup2000bp to generate pGL4.12-hMUC5ACup3000bp. To delete the 15 bp in the *MUC5AC* promoter region (125 bp to 111 bp upstream), PCR-based mutagenesis was performed using primers MUC5ACup-15delete-F and MUC5ACup-15delete-R and cloned into pGL4.12 to generate pGL4.12-hMUC5ACup451bp-delta15bp. The plasmid was confirmed by sequence analysis. The 284 bp SbfI-HindIII region of pGL4.12-hMUC5ACup4010bp was replaced by the 269 bp SbfI-HindIII fragment of pGL4.12-hMUC5ACup451bp-delta15bp to generate pGL4.12-hMUC5ACup4010bp-delta15bp.

**Table 1:** Primers used for plasmid constructions.

Name	Sequence
MUC5ACup-F-01	5′-attcattcacccattcactcactcattcac-3′
MUC5ACup-F-02	5′-tgccacatgtgaagtgctctttctctaggc-3′
MUC5ACup-R-01	5′-tgtgtggacggcggggaagagtgccctgtc-3′
MUC5ACup-280-XhoI	5′-taggctcgagtatgtggggaggacccctgc-3′
MUC5ACup-190-XhoI	5′-cgccctcgagcggccgctggccagcccgca-3′
MUC5ACup-158-XhoI	5′-gagcctcgagtgtttacttgggtgaggggg-3′
MUC5ACup-110-XhoI	5′-gcccctcgaggtgaagcacggggctggagc-3′
MUC5ACup-150-XhoI	5′-actgctcgagtgggtgagggggaaccacag-3′
MUC5ACup-138-XhoI	5′-ggtgctcgagaaccacaggccccgccctgc-3′
MUC5ACup-125-XhoI	5′-cacactcgaggccctgcccacccacgtgaa-3′
pGL412-HindIII	5′-ccggattgccaagcttggcc-3′
MUC5ACup-SacI-F	5′-tgcccacagagctcagaaacaaggc-3′
MUC5ACup-PciI-R	5′-agagcacttcacatgtggcaggagt-3′
MUC5ACup-3000-NheI-F	5′-acttgctagctcatacactcattcacttat-3′
MUC5ACup-15delete-F	5′-gagggggaaccacaggccccgtgaagcacggggctggagc-3′
MUC5ACup-15delete-R	5′-gctccagccccgtgcttcacggggcctgtggttccccctc-3′

Gli1 and Gli3 cDNAs were purchased from Open Biosystems (clone ID 3531657 and 40125719, respectively, Huntsville, AL,) and Gli2 cDNA (pENTR223.1-hGli2) was purchased from DNAFORM K.K. (clone ID 100069128, Yokohama, Japan). EcoRI-XhoI fragment containing Gli1 cDNA was inserted into the EcoRI/XhoI site of pMXs-IP to generate pMXs-Gli1-IP. EcoRI-EcoRV fragment containing Gli3 cDNA was inserted into the EcoRI/PacI site of pMXs-IP to generate pMXs-Gli3-IP. XbaI-NotI fragments from pMXs-Gli1-IP and pMXs-Gli3-IP were inserted into the NheI/NotI site of pcDNA3.1(+) to generate pcDNA3.1(+)-Gli1 and pcDNA3.1(+)-Gli3. BsrGI-AvrII fragment from pENTR223.1-hGli2 was inserted into the Acc65I/XbaI site of pcDNA3.1(+) to generate pcDNA3.1(+)-Gli2.

### Luciferase reporter assay

Cells were cultured on 96-well plates and transiently transfected with mixtures of Renilla luciferase control vector pGL4.74 (Promega) (15 ng) and the firefly luciferase reporter plasmids (150 ng) by using Lipofectamine and Lipofectamine PLUS (Invitrogen). To examine the effect of Gli on the promoter activities, pcDNA3.1(+)-Gli1, -Gli2, and -Gli3 plasmids were transfected with pGL-based plasmids at the same time. Luciferase assays were performed at 24 or 48 h post-transfected using the Dual luciferase reporter assay system (Promega). Luciferase activities were measured by an AutoLumat Plus LB953 (Berthold, Bad Wildbad, Germany). Luciferase activity was normalized to a Renilla control and the results are shown as mean ± SD from at least three independent experiments.

### Production and infection of retrovirus vectors

pMXs-Gli1-IRES-Puro, pMXs-Gli2-IRES-Puro and pMXs-Gli3-IRES-Puro and control vector (pMXs-IRES-Puro) were transfected with pCAG-VSVG into the PLAT-GP packaging cell line for retrovirus vector production (Cell Biolabs, Inc. San Diego, CA) using Lipofectamine and Lipofectamine PLUS (Invitrogen). Virus-containing supernatants were harvested at 24 h, 48 h and 72 h post-transfection, pooled, and filtered though a 0.45-µm filter (Millipore, Bedford, MA). To assess the effects of overproduction of Gli, cells were infected by adding retrovirus-containing supernatant at a final concentration of 8 µg/ml Polybrene (Sigma) and were selected over 3 days with puromycin (Sigma).

### Transfection

Cells were transiently transfected with pcDNA3.1(+)-Gli1, pcDNA3.1(+)-Gli2, pcDNA3.1(+)-Gli3 and pcDNA3.1(+) plasmids for 24–48 h using Lipofectamine and Lipofectamine PLUS, and were used for RT-PCR and luciferase reporter assay.

### RT-PCR

Total cellular RNA was prepared using the Isogen RNA isolation reagent (Wako Pure Chemical Industries, Osaka, Japan) as previously reported [29]. RT-PCR was performed via a Superscript One-Step reaction using the Platinum Taq (Invitrogen). The primer sequences used for RT-PCR are shown in Table 2. RNA was reverse-transcribed for 30 min at 50°C, and after an initial denaturation at 94°C for 3 min, cDNA amplification procedures were performed as follows: for *MUC5AC*, 35 cycles of 94°C for 30 sec, 64°C for 1 min, and 72°C for 1 min; for *GAPDH*, 30 cycles of 94°C for 30 sec, 60°C for 1 min, and 72°C for 1 min; for *Gli1* and *Gli2*, 35 cycles of 94°C for 30 sec, 60°C for 1 min, and 72°C for 1 min; for *Gli3*, 40 cycles of 94°C for 30 sec. A commercial RNA panel, Human Total RNA Master Panel II, was purchased from Clontech Laboratories (Palo Alto, CA, USA).

**Table 2:** Primer pairs, annealing temperatures (Tm), and product sizes (Length) for the 5 genes analyzed by RT-PCR.

Genes		Forward (F) and reverse (R) primer sequences	Tm(°C)	Length
MUC5AC	F	5′-accggtgccacatgacggac-3′	64	396
	R	5′-acgtggccgcctcacacgtg-3′		
Gli1	F	5′-gaaggagttcgtgtgccact-3′	60	491
	R	5′-gtctgctttcctccctgatg-3′		
Gli2	F	5′-gcaacaaagccttctccaac-3′	60	430
	R	5′-atctccacgccactgtcatt-3′		
Gli3	F	5′-aaagcaaacaggagcctgaa-3′	60	401
	R	5′-ggaatgcgttctgttttggt-3′		
GAPDH	F	5′-accacagtccatgccatcac-3′	60	423
	R	5′-tccaccaccctgttgctgta-3′		

### siRNA

SH-10-TC cells were transfected with siGENOME SMARTpool siRNA against human Gli1 and/or Gli2 (M-003896-00-0005 and M-006468-02-0005, respectively; Dharmacon, Lafayetta, CO, USA) using Lipofectamine RNAiMAX (Invitrogen) according to the manufacture’s instructions. RNA was extracted from the cells 48 h after siRNA transfection and used for RT-PCR.

### Chromatin immunoprecipitation (ChIP) assay

ChIP analyses were performed using a ChIP assay kit (Upstate Biotechnology Inc., Lake Placid, NY) according to the manufacture’s instructions. For crosslinking, cells (2×10^6^) were incubated at 37°C for 15 min in PBS containing 1% formaldehyde. Cells were collected after two washes with ice-cold PBS and subjected to centrifugation for 5 min at 700×g. Cells pellets were resuspended 400 µl sodium dodecyl sulfate (SDS) lysis buffer (ChIP assay kit, Upstate) containing protease inhibitors (0.1 M phenylmethylsulfonylfluoride and 4 µl protease inhibitor cocktail (Sigma)), and incubated for 10 min on ice. DNA was sonicated (setting 5, Handy Sonic, model UR-20P; Tomy Seiko, Co., Ltd., Tokyo, Japan) and then subjected to centrifugation at 13,000 rpm for 10 min. Supernatants were diluted 10-fold with Chip dilution buffer (ChIP assay kit, Upstate). 1% of the supernatant was retained as the input, and the rest was then subjected to immunoprecipitations. Immunoprecipitations were performed overnight at 4°C with 3 µg of anti-Gli1 antibody (H-300, sc-20687; Santa Cruz Biotechnology, Santa Cruz, CA) and nonimmunized rabbit IgG whole molecule (sc-2027; Santa Cruz Biotechnology). After reverse crosslinking, the obtained DNA was purified using PCR product purification kit (Qiagen, Valencia, CA, USA). Immunoprecipitated DNA was analyzed by PCR using primers 5′-ctcggaaactgggctctacccgg-3′ and 5′-gagctttttgtagccccagagctgg-3′ to amplify a fragment of the *MUC5AC* promoter region. Primer pairs for the *villin 1* promoter region were used as a negative control [8].

## Results

### Decrease of MUC5AC expression accompanied with progression of gastric canceration

**Figure 1:**
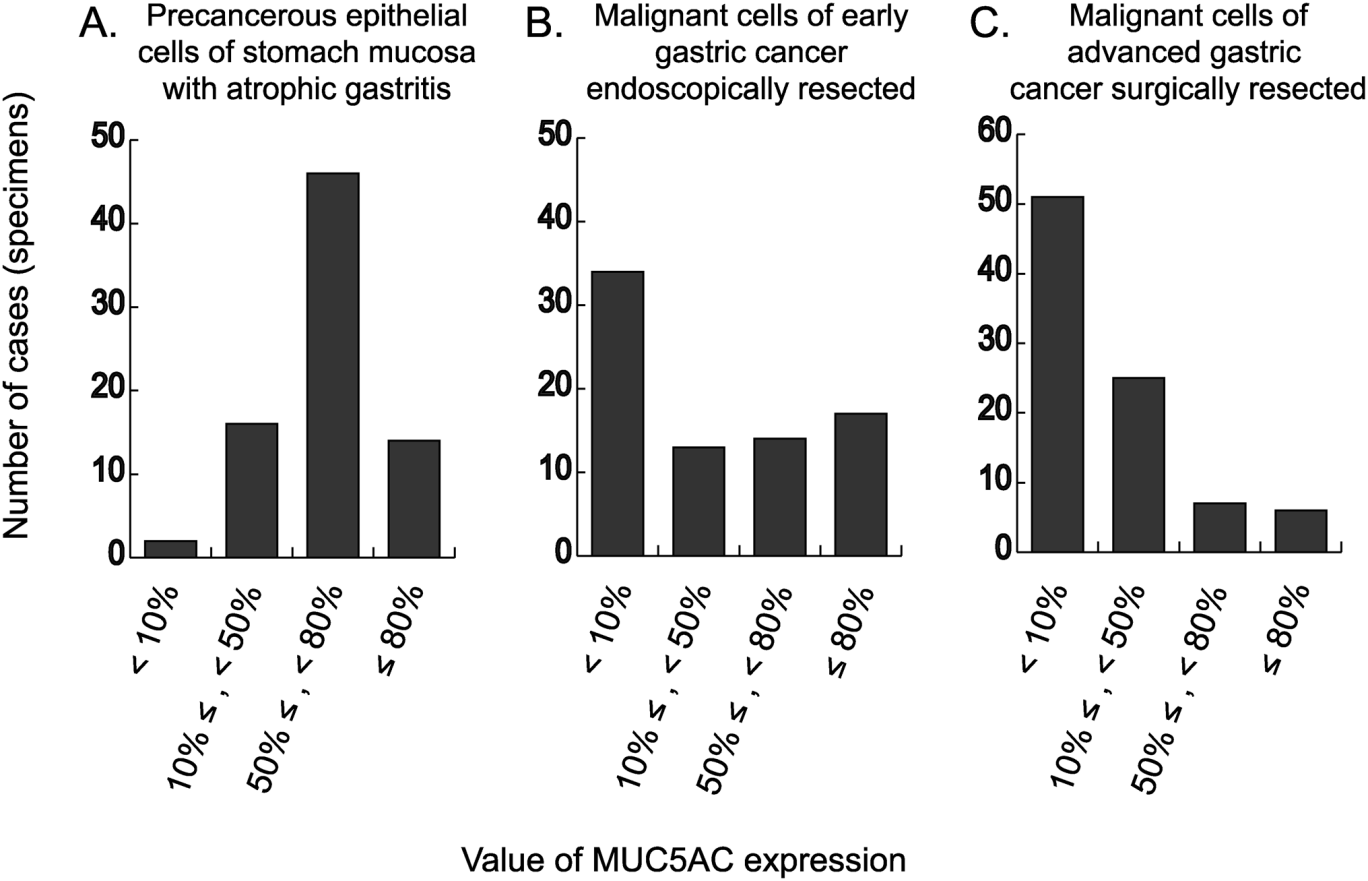
Expression of MUC5AC in non-tumorous epithelial cells of atrophic mucosa around early gastric cancer endoscopically resected (A), malignant cells of early gastric cancer endoscopically resected (B), and malignant cells of advanced gastric cancer surgically resected (C). Based on the percentages of cells with immunoreactivity of MUC5AC, the 78 non-malignant or malignant specimens (A, B) and 89 malignant specimens (C) were classified into four categories: 1) <10%, 2) ≥10% and <50%, 3) ≥50% and <80%, and 4) ≥80%.

To evaluate the expression of MUC5AC in gastric cancer, immunostaining was performed using the clinical specimen representing three-grade gastric epithelial cells from the view of tumorigenesis: non-tumorous but precancerous cells of atrophic mucosa around early gastric cancer (Fig. 1A), malignant cells of early gastric cancer endoscopically resected (Fig. 1B), and malignant cells of advanced gastric cancer surgically resected (Fig. 1C). Our results revealed that the stronger the malignant property of gastric epithelial cells is, the weaker the expression of MUC5AC is. In other word, expression of MUC5AC decreases accompanied with increased malignant property of gastric mucosa.

Some previous studies including ours reported that expression of MUC5AC often attenuates during the development of pre-malignant intestinal metaplasia [6], [14]. Our present data further showed that decrease of MUC5AC expression advances in the process of gastric canceration. From these results, we are convinced that it is important to elucidate the regulatory mechanism of *MUC5AC* gene expression for understanding tumorigenesis of gastric cancer.

### A highly conserved region containing a Gli-binding sequence (HCR-Gli) is present in the promoter of *MUC5AC* gene

**Figure 2:**
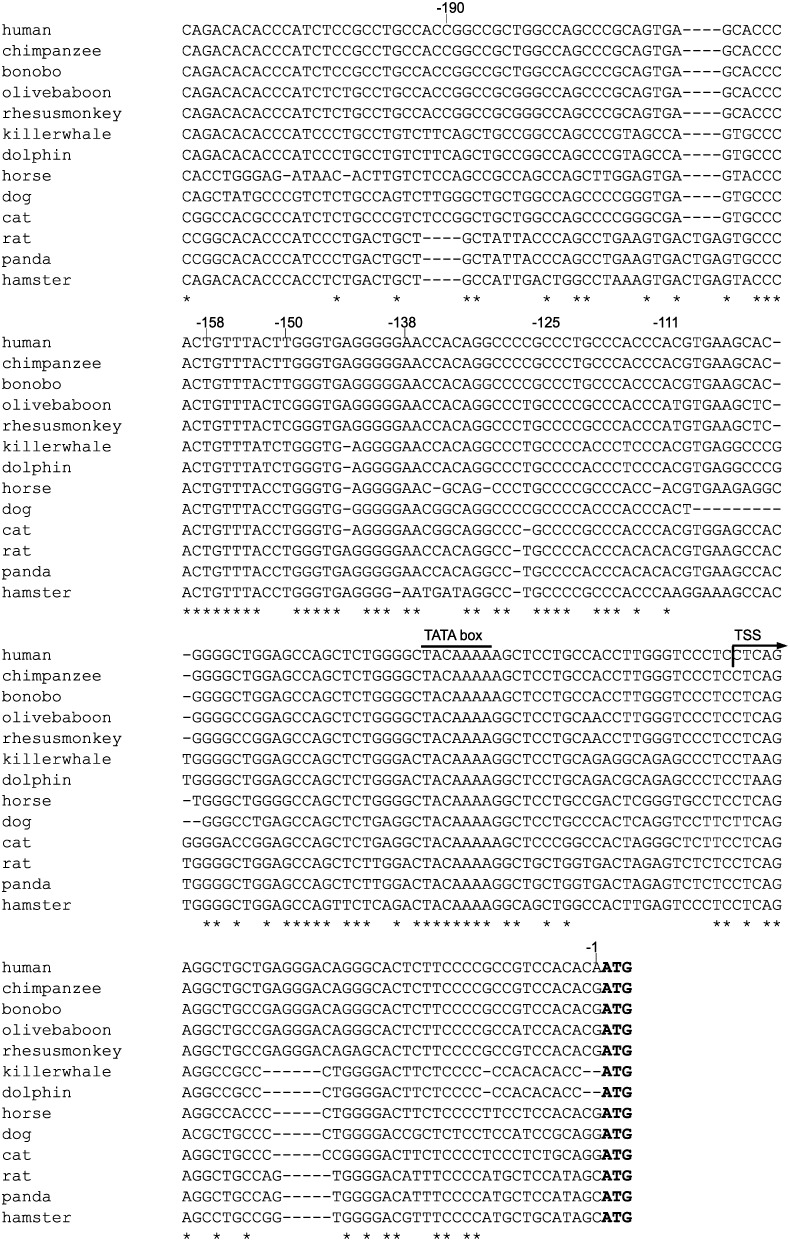
Comparison of the 5′-upstream sequences of *MUC5AC* gene of 13 mammal species. Alignment was carried out using the ClustalW (ver 2.1). The translational start site (TSS, arrow) and the translational starting codon (ATG, bold) are indicated. Asterisks (*) represent exact matches in all the sequences.

To examine the transcriptional regulation of *MUC5AC* gene, the upstream 5′-flanking regions of *MUC5AC* gene of 13 mammal species retrieved from GenBank (dated 8 May, 2013) were aligned using ClustalW (ver 2.1) sequence alignment program [30]. The major transcription initiation site of human *MUC5AC* gene has been mapped to 48 bp upstream of the ATG translation start site [31]. Alignment of the human sequence with other mammal sequences denoted high homology among orthologs within ∼200 bp upstream of the ATG start codon (Fig. 2).

**Figure 3:**
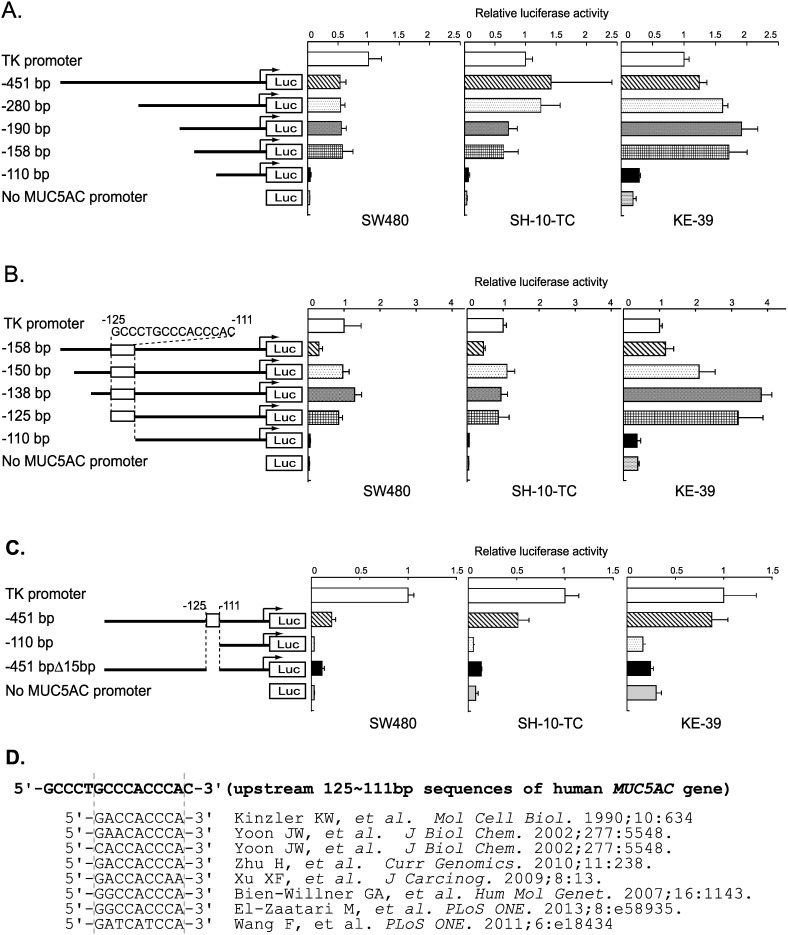
Luciferase reporter analyses of a series of *MUC5AC* promoter constructs in gastrointestinal cell lines and identified highly conserved sequence similar to known Gli-binding sequences. (A–C) Data represent the mean of luciferase activities of SW480, SH-10-TC, and KE-39 cells measured at 24 h after transfection. The error bars showed the standard deviation of the results from three independent experiments. (D) Alignment of the highly conserved sequence in *MUC5AC* promoter region with previous reported sequences of Gli-binding site.

To test the function of the conserved promoter regions identified above, a series of luciferase reporter constructs containing 451 bp or shorter fragments of the human *MUC5AC* promoter region were generated. Promoter activities of these deletion constructs were measured by luciferase reporter assay in the three *MUC5AC*-expressing cell lines derived from human gastrointestinal cancer: SW480, SH-10-TC, and KE-39 [6]. As shown in Fig. 3A, the promoter activity of the reporter construct containing upstream 110 bp was much lower than those containing upstream 451, 280, 190, and 158 bp.

To examine the essential region for the luciferase activity between −158 to −110 bp more precisely, reporter constructs containing 150, 138, and 125 bp upstream of ATG start codon were generated and examined their activities (Fig. 3B). Although the reporter constructs containing upstream 158, 150, 138, and 125 bp showed high luciferase activities, the construct containing upstream 110 bp denoted very low activities equal to a negative control.

Next we made a deletion mutant construct containing upstream 451 bp but lacking the region between −125 and −111 bp, and examined the promoter activity (Fig. 3C). Deletion of the 15 bp led to a marked decrease in the luciferase activity, suggesting that the 15 bp sequence between −111 to −125 bp is critical for the *MUC5AC* promoter activity. Based on the thorough literature search [32], [33], [34], [35], [36], [37], [38], we found a putative Gli-binding site in this short 15 bp sequence (5′-GCCCTGCCCACCCAC-3′ shown in Fig. 3D). We could not find any other transcription factors which bind to this 15 bp sequence by searching transcription factor database. Consequently, we named this 15 bp sequence as a highly conserved region containing a Gli-binding site (HCR-Gli).

### Exogenous modulation of Gli affects the endogenous *MUC5AC* gene expression in gastrointestinal cells

**Figure 4:**
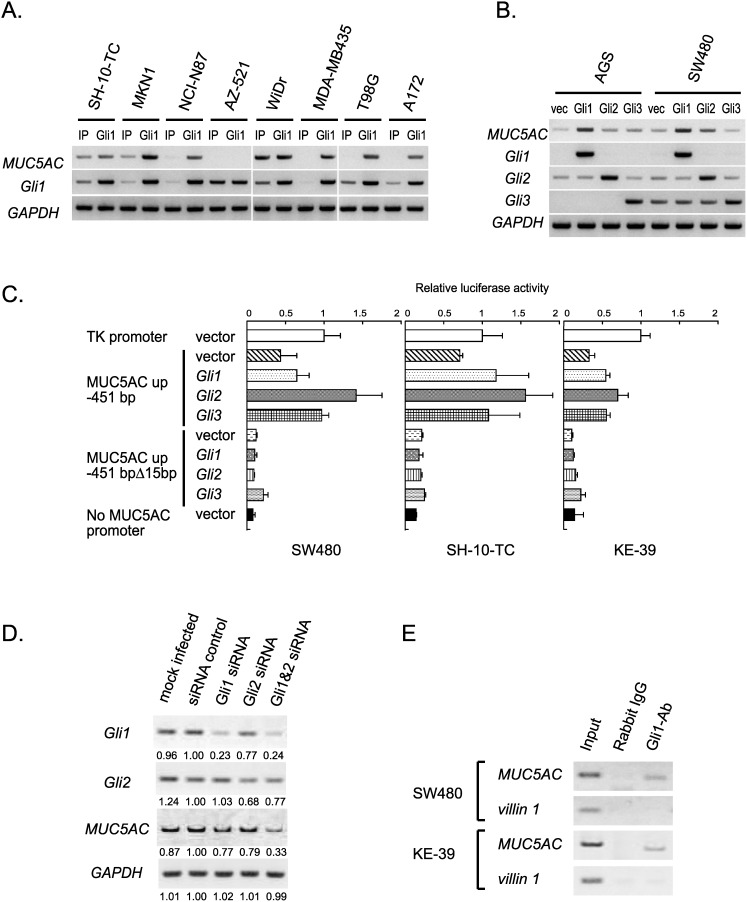
Effects of exogenous modulation of Gli on *MUC5AC* gene expression. (A) RT-PCR detecting *MUC5AC* mRNA in 5 gastrointestinal and 3 non-gastrointestinal cell lines infected with retroviral vector encoding *Gli1* gene. (B) RT-PCR detecting *MUC5AC* mRNA in AGS and SW480 cells transfected with pcDNA3.1(+) (vec), pcDNA3.1(+)-Gli1, pcDNA3.1(+)-Gli2 or pcDNA3.1(+)-Gli3. (C) Luciferase reporter analysis of *MUC5AC* promoter constructs in SW480, SH-10-TC, and KE-39 cells transfected with pcDNA3.1(+) (vector), pcDNA3.1(+)-Gli1, pcDNA3.1(+)-Gli2 or pcDNA3.1(+)-Gli3. (D) *MUC5AC* expression analyzed by RT-PCR using SH-10-TC cells transfected with control siRNA, Gli1 siRNA alone, Gli2 siRNA alone, or Gli1 and Gli2 siRNA. (E) ChIP analyses of the human *MUC5AC* gene in SW480 and KE-39 cells. Anti-Gli1 antibody (Gli1-Ab) and non-immunized rabbit IgG whole molecule were used for immunoprecipitation. PCR was performed with primers recognizing the HCR-Gli-containing fragment of *MUC5AC* promoter or recognizing the promoter region of *villin1* gene.

We examined whether Gli1 can upregulate *MUC5AC* transcription activity in gastrointestinal cell lines. We constructed a retrovirus vectors carrying the *Gli1* gene, and transduced it into four gastric (SH-10-TC, MKN1, NCI-N87, AZ-521), one colorectal (WiDr), and three other cancer cell lines (MDA-MB435, T98G, A172). In the resulting stable transductants, the expression levels of *MUC5AC*, *Gli1*, and *GAPDH* were analyzed by RT-PCR (Fig. 4A). Although there were two exceptions of AZ-521 and WiDr, *MUC5AC* was found to be universally upregulated by Gli1 overexpression.

We next compared the three Gli homologs (*Gli1*, *Gli2*, and *Gli3*) identified in human. To examine the effect of these transcription factors on *MUC5AC* expression, AGS and SW480 cell lines transiently overexpressing Gli1, Gli2 and Gli3 were analyzed by RT-PCR (Fig. 4B). Gli1 strongly upregulated *MUC5AC* expression, which was consistent with the result of stable transductants in Fig. 4A. Gli2 and Gli3 also increased the *MUC5AC* expression, but their effects were weaker than Gli1.

To address whether Gli1, Gli2, and Gli3 activate the *MUC5AC* promoter through the 15 bp of HCR-Gli, we performed the luciferase reporter assay using the construct containing upstream 451 bp of the human *MUC5AC* gene and the deletion mutant lacking the HCR-Gli (Fig. 4C). In the *Gli*-transfected SW480, SH-10-TC, and KE-39 cells, all the three Gli homologs enhanced activation of the reporter construct containing upstream 451 bp of *MUC5AC*, but not of the deletion mutant lacking the HCR-Gli. These results indicate that Gli1, Gli2, and Gli3 can efficiently activate *MUC5AC* transcription through the HCR-Gli. From these results, we speculate that Gli1, Gli2 and Gli3 may play redundant roles but the effect of Gli1 is stronger than that of others upon the upregulation of *MUC5AC* expression.

We next examined the effect of Gli knockdown by siRNA transfection on *MUC5AC* expression. After SH-10-TC cells were transfected with Gli1 and/or Gli2 siRNA, the expression levels of *MUC5AC*, *Gli1*, *Gli2*, and *GAPDH* were analyzed by RT-PCR. As shown in Fig. 4D, double knockdown of Gli1 and Gli2 most efficiently decreased *MUC5AC* expression, whereas the effects of either Gli1 or Gli2 single knockdown were considerably weaker. These results suggest that Gli1 and Gli2 have redundant functions for *MUC5AC* expression, which is consistent with our above-mentioned speculation.

### Gli interacts to HCR-Gli in the *MUC5AC* promoter region

To determine whether Gli1 directly binds to the *MUC5AC* promoter, chromatin immunoprecipitation (ChIP) assays were performed using anti-Gli1 antibody. As a negative control, the human *villin1* promoter sequence was used because *villin1* is a typical intestinal marker gene regulated by Cdx [8], and also because exogenous modulation of Gli1 expression level did not change endogenous *villin1* expression (our unpublished observation). As shown in Fig. 4E, the HCR-Gli-containing fragment of *MUC5AC* promoter was co-immunoprecipitated with Gli1 in SW480 and KE-39 cells, whereas the upstream sequence of *villin1* promoter was not. Our results demonstrate that Gli1 activates *MUC5AC* gene transcription through direct interaction between Gli1 and the *MUC5AC* promoter region.

Taking all the results from luciferase assays with upstream deletion constructs, overexpression and knockdown of Gli, and ChIP analyses into consideration, we concluded that Gli enhances the *MUC5AC* gene transcription via direct protein-DNA interaction on the HCR-Gli region.

### Gli is not sufficient for transcription of *MUC5AC* gene in gastrointestinal cells

**Figure 5:**
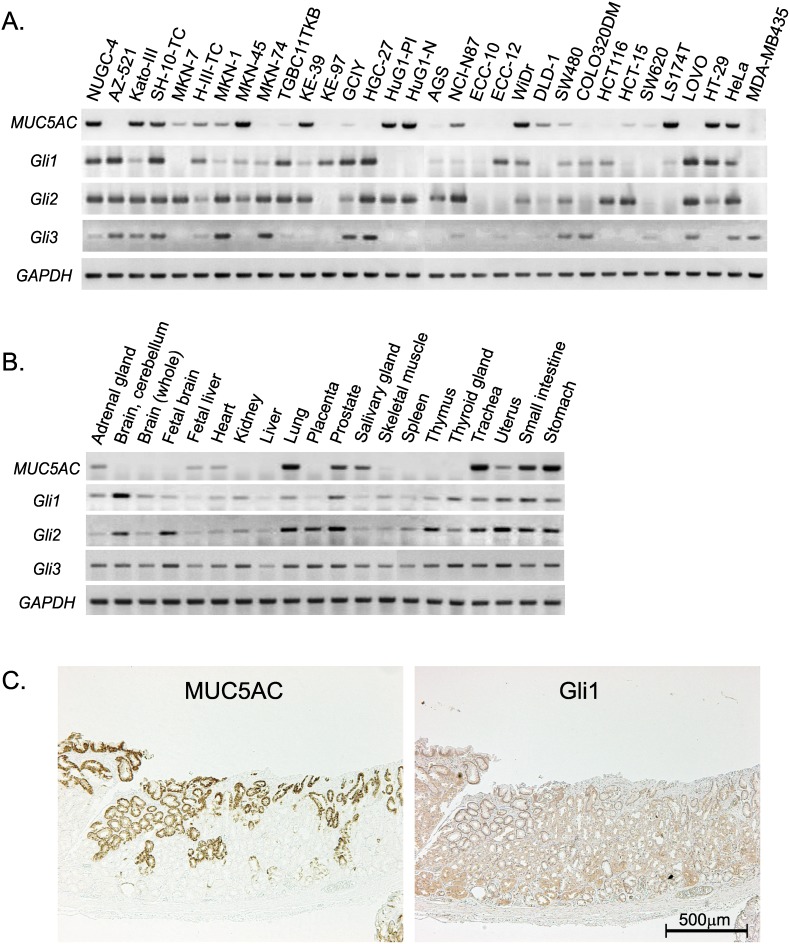
Expression profiles of Gli and MUC5AC in various tumor cell lines and human normal tissues. (A, B) RT-PCR detecting *MUC5AC* and *Gli* homologs in the 30 tumor cell lines (A) and systemic normal tissues (B). (C) Immunostaining of MUC5AC (left panel) and Gli1 (right panel) in sequential sections of non-malignant gastric mucosa.

To examine the relationship between endogenous expression levels of *Gli* and *MUC5AC* in gastrointestinal cells, RT-PCR was performed using the twenty gastric, ten colorectal, and two non-gastrointestinal cancer cell lines (Fig. 5A). All the cell lines with endogenous *MUC5AC* expression were found to be positive for *Gli1* and/or *Gli2* expression. Conversely, there were several cell lines in which *Gli1* and/or *Gli2* were expressed whereas *MUC5AC* was not.

Tissue expression patterns of *MUC5AC* and *Gli* were also examined using a commercially available RNA panel of normal human tissues (Fig. 5B). Although several adult tissues such as stomach, lung, trachea, etc. obviously expressed *MUC5AC* mRNA, about half of the analyzed tissues were deficient in *MUC5AC* expression. On the other hand, *Gli1*, *Gli2*, and *Gli3* were ubiquitously expressed in almost all the examined tissues. It should be noted that *MUC5AC* expression were not detected in adult brain, kidney, liver, placenta, skeletal muscle, spleen, thymus, thyroid gland, etc., despite the expression of the three *Gli* homolog genes (Fig. 5B).

Using non-malignant gastric tissues derived from the surgical specimens, we further evaluated the protein expression of MUC5AC and Gli1 by immunohistochemistry. As shown in Fig. 5C, Gli1 was weakly but ubiquitously expressed on gastric mucosa, which was different from the spotty immunostaining of MUC5AC. All the gastric tissues derived from the seven surgical specimens showed the same expression pattern.

In total, expression patterns of MUC5AC and Gli clearly support our conclusion that Gli is necessary for *MUC5AC* expression in human gastrointestinal cells. In addition, it is also obvious that expression of Gli is not enough for *MUC5AC* expression, suggesting that other mechanism should work cooperatively on the transcription of *MUC5AC* gene.

### Multiple regulatory mechanisms work in the promoter region of *MUC5AC* gene

**Figure 6:**
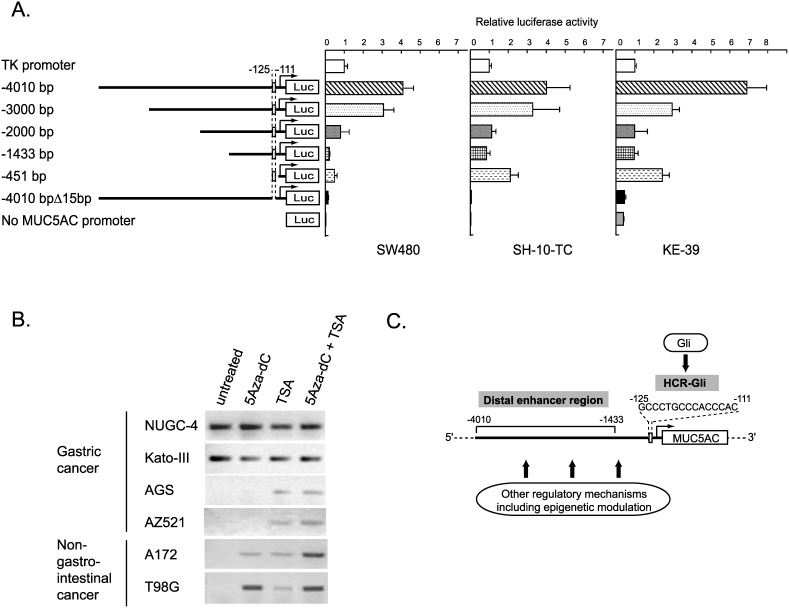
Multiple regulatory mechanisms of *MUC5AC* gene expression. (A) Luciferase reporter analysis of a series of constructs covering the far upstream sequence in the *MUC5AC* promoter. Data represent the mean of luciferase activities in SW480, SH-10-TC, and KE-39 cells measured at 24 h after transfection. The error bars showed the standard deviation of the results from three independent experiments. (B) Expression of *MUC5AC* analyzed by RT-PCR, using the 4 gastric cancer-derived and 2 glioblastoma-derived cell lines treated with 5-Aza-2′-deoxycytidine (5-Aza-dC) and/or trichostatin A (TSA). (C) Schematic representation of the presumed multiple regulations of human *MUC5AC* gene.

To analyze the far distal promoter region of *MUC5AC* gene, we generated reporter constructs containing 4010, 3000, 2000, and 1433 bp upstream of ATG start codon and examined their promoter activities (Fig. 6A). Deletion from 4010 bp to 1433 bp region revealed gradual decrease in transcriptional activity, suggesting the presence of enhancer elements in this distal promoter region. However, even in the reporter construct including long 4010 bp, deletion of the 15 bp HCR-Gli region resulted in the drastic decrease of luciferase activity (Fig. 6A). Together, our results demonstrated that there are at least two regulatory elements in the *MUC5AC* promoter region: the 15 bp sequence between −111 bp to −125 bp which critical for the promoter activity, and the wider regulatory region in −4010 bp to −1433 bp which can enhance the promoter activity.

To further examine the possible epigenetic regulation on *MUC5AC* expression, four gastrointestinal cell lines and two glioblastoma cell lines were treated with demethylating agent (5Aza-dC) and/or HDAC inhibitor (TSA). Transcription of *MUC5AC* was slightly upregulated in all the four *MUC5AC*-deficient cell lines (Fig. 6B), suggesting that epigenetic regulation such as methylation and/or histone acetylation additionally works as the regulatory mechanism of *MUC5AC* expression. From the results of four *MUC5AC*-deficient cell lines, methylation seems to play some universal role on suppression of *MUC5AC* expression. Synergistic effects of 5-Aza-dC and TSA in A172 and T98G cells also suggested that modification of histone acetylation may have some influence on *MUC5AC* transcription.

## Discussion

MUC5AC, a secreted mucin highly detected in the superficial gastric epithelium, is a well-known gastric marker often used for classification of stomach cancer [20], [39]. We have previously reported that expression of *MUC5AC* in stomach decreases in association with development of intestinal metaplasia [6]. In the present study, we further showed that *MUC5AC* expression is apparently related to the tumor stage: advanced gastric cancers present reduced levels of MUC5AC compared with early gastric cancer. Despite the anticipated importance of *MUC5AC* regulation, the molecular mechanisms underlying *MUC5AC* expression in gastrointestinal cells remain poorly understood. In this study, we searched 5′-upstream of *MUC5AC* gene and identified the HCR-Gli at −125/−111 bp in its promoter region. Our overexpression/knockdown analyses and luciferase reporter assays revealed that Gli induced *MUC5AC* expression through the HCR-Gli in gastrointestinal cells. In addition, ChIP analysis showed that Gli1 directly binds to HCR-Gli. The results of RT-PCR using 30 gastrointestinal tumor cell lines and an RNA panel from systemic normal tissues suggested that Gli is necessary but not sufficient for *MUC5AC* expression, which was consistent with the results from immunohistochemistry of clinical specimens. Furthermore, we found that the far distal upstream region from −1433 to −4010 bp enhances the Gli-dependent *MUC5AC* expression, at least partly based on the epigenetic modulation.

Recently, Inaguma *et al.* reported that Gli1 activates the *MUC5AC* promoter through two putative Gli-binding sites (GBS1 and GBS2) in pancreatic cancer cells [25]. However, the significance of *MUC5AC* expression is different in gastrointestinal cells and pancreatic cells. Namely, abundant expression of MUC5AC is observed in normal epithelial cells of stomach but its expression is decreased accompanied with development of intestinal metaplasia [14], [20]. On the other hand, *MUC5AC* is generally undetectable in normal pancreas tissue and is sometimes ectopically induced in pancreatic tumor cells. In fact, there are two differences between Inaguma’s report and our results. First, Inaguma *et al.* showed that both GBS1 and GBS2 respond to upregulation of *MUC5AC* expression by Gli. The GBS1 overlaps to our identified HCR-Gli, but our results indicated that the GBS2 (corresponding to −159/−168 bp in Fig. 2) has no effect on *MUC5AC* expression (Fig. 3A). Second, Inaguma *et al.* reported that Gli1 expression correlates with MUC5AC induction by immunohistochemistry. However, our results revealed that expression of Gli does not always accompany with *MUC5AC* expression in gastrointestinal cells (Figure 5). These inconsistencies might be due to a difference of originated tissues (pancreatic or gastrointestinal).

No induction of *MUC5AC* expression in AZ521 cells (Fig. 4A), which completely lack the SWI/SNF complex-based chromatin remodeling activity [28], may suggest the interaction between Gli and the SWI/SNF complex. Although there have been no previous reports concerning the interaction with Gli, SWI/SNF chromatin remodeling complex has been reported to bind with various transcription factors such as AP-1 [40], CREB [41], MyoD [42], Cdx [8], GATA1 [43], and so on.

The 5′-upstream distal region from −1433 to −4010 bp in *MUC5AC* promoter showed obvious enhancing effect on *MUC5AC* expression (Fig. 6A). Interestingly, we noticed that the region from −1455 to −3187 bp contains 86 copies of tandem sequences 5′-TCA(C/T)TCA(C/T)-3′ (Fig. 6C) [44]. The tandem repeats are conserved among mammals especially among primate and cetartiodactyla. Human, olive baboon, rhesusmonkey, killerwhale, and dolphin have more than 60 repeats of the tandem repeats in their 4010 bp upstream of MUC5AC promoter region. In contrast, horse, dog, cat, panda, and hamster have only less than 10 repeats of the tandem repeats. We have no information for this tandem repeat sequences at present, but it might has some roles on regulation of *MUC5AC* expression.

Recently, Yamada *et al.* showed that the CpG methylation status of around −3.7 kb in the *MUC5AC* promoter is associated with *MUC5AC* expression in some cancer cell lines, suggesting that the expression of *MUC5AC* is epigenetically regulated in the distal promoter region [45]. In fact, we found increase in *MUC5AC* expression by 5Aza-dC and/or TSA treatment, indicating some epigenetic mechanism involved in *MUC5AC* gene regulation (Fig. 6C).

To understand gastric tumorigenesis mainly occurred in the atrophic mucosa with chronic gastritis and intestinal metaplasia, we believe the disrupted balance between intestinal and gastric differentiation must be important [8], [28]. For intestinal differentiation, many previous reports indicated that Cdx must be an indispensable key molecule based on the transcriptional regulation of many intestinal differentiation marker genes [8], [9], [10], [11], [12]. On the contrary, the critical key molecule for gastric differentiation has not been identified yet. Despite the through screening, we have not found the essential regulator for our identified gastric marker *Cathepsin E* (data not shown) [6], and we have elucidated that typical gastric marker *MUC5AC* gene is partly regulated by Gli, a universal transcription factor which alone cannot realize the tissue-specific expression of *MUC5AC* gene. Unlike intestinal differentiation, we now speculate that gastric differentiation may depend on cooperative regulatory mechanism of some universal transcription factors and epigenetic modulations.

In summary, the present study has shown that Gli regulates *MUC5AC* gene expression via direct protein-DNA interaction through the highly conserved 15 bp sequence between −125 and −111 bp in the promoter region of *MUC5AC*. Furthermore, immunohistochemical analysis and RT-PCR using systemic normal tissue revealed that Gli is necessary but not sufficient for *MUC5AC* expression. We conclude that *MUC5AC* expression is regulated by combination of multiple regulatory mechanisms such as universal transcription factors and epigenetic modulations.

## References

[1] Gonzalez CA, Agudo A (2012) Carcinogenesis, prevention and early detection of gastric cancer: where we are and where we should go. Int J Cancer 130: 745–753. 21918974 10.1002/ijc.26430

[2] Leung WK, Sung JJ (2002) Review article: intestinal metaplasia and gastric carcinogenesis. Aliment Pharmacol Ther 16: 1209–1216. 12144569 10.1046/j.1365-2036.2002.01300.x

[3] Polk DB, Peek RM Jr (2010) Helicobacter pylori: gastric cancer and beyond. Nat Rev Cancer 10: 403–414. 20495574 10.1038/nrc2857 PMC2957472

[4] Tsukamoto T, Mizoshita T, Tatematsu M (2006) Gastric-and-intestinal mixed-type intestinal metaplasia: aberrant expression of transcription factors and stem cell intestinalization. Gastric Cancer 9: 156–166. 16952033 10.1007/s10120-006-0375-6

[5] Filipe MI, Munoz N, Matko I, Kato I, Pompe-Kirn V, et al (1994) Intestinal metaplasia types and the risk of gastric cancer: a cohort study in Slovenia. Int J Cancer 57: 324–329. 8168991 10.1002/ijc.2910570306

[6] Konno-Shimizu M, Yamamichi N, Inada K-i, Kageyama-Yahara N, Shiogama K, et al (2013) Cathepsin E Is a Marker of Gastric Differentiation and Signet-Ring Cell Carcinoma of Stomach: A Novel Suggestion on Gastric Tumorigenesis. PLoS ONE 8: e56766. 23451082 10.1371/journal.pone.0056766 PMC3579941

[7] Yuasa Y (2003) Control of gut differentiation and intestinal-type gastric carcinogenesis. Nat Rev Cancer 3: 592–600. 12894247 10.1038/nrc1141

[8] Yamamichi N, Inada K, Furukawa C, Sakurai K, Tando T, et al (2009) Cdx2 and the Brm-type SWI/SNF complex cooperatively regulate villin expression in gastrointestinal cells. Exp Cell Res 315: 1779–1789. 19371634 10.1016/j.yexcr.2009.01.006

[9] Mesquita P, Jonckheere N, Almeida R, Ducourouble MP, Serpa J, et al (2003) Human MUC2 mucin gene is transcriptionally regulated by Cdx homeodomain proteins in gastrointestinal carcinoma cell lines. J Biol Chem 278: 51549–51556. 14525978 10.1074/jbc.M309019200

[10] Hinoi T, Lucas PC, Kuick R, Hanash S, Cho KR, et al (2002) CDX2 regulates liver intestine-cadherin expression in normal and malignant colon epithelium and intestinal metaplasia. Gastroenterology 123: 1565–1577. 12404231 10.1053/gast.2002.36598

[11] Suh E, Chen L, Taylor J, Traber PG (1994) A homeodomain protein related to caudal regulates intestine-specific gene transcription. Mol Cell Biol 14: 7340–7351. 7935448 10.1128/mcb.14.11.7340-7351.1994 PMC359269

[12] Park J, Schulz S, Waldman SA (2000) Intestine-specific activity of the human guanylyl cyclase C promoter is regulated by Cdx2. Gastroenterology 119: 89–96. 10889158 10.1053/gast.2000.8520

[13] Barros R, da Costa LT, Pinto-de-Sousa J, Duluc I, Freund JN, et al (2012) CDX2 autoregulation in human intestinal metaplasia of the stomach: impact on the stability of the phenotype. Gut 60: 290–298. 10.1136/gut.2010.222323 PMC3034084 21148572

[14] Reis CA, David L, Nielsen PA, Clausen H, Mirgorodskaya K, et al (1997) Immunohistochemical study of MUC5AC expression in human gastric carcinomas using a novel monoclonal antibody. Int J Cancer 74: 112–121. 9036879 10.1002/(sici)1097-0215(19970220)74:1<112::aid-ijc19>3.0.co;2-h

[15] Wakatsuki K, Yamada Y, Narikiyo M, Ueno M, Takayama T, et al (2008) Clinicopathological and prognostic significance of mucin phenotype in gastric cancer. J Surg Oncol 98: 124–129. 18521835 10.1002/jso.21093

[16] Kim SM, Kwon CH, Shin N, Park do Y, Moon HJ, et al (2014) Decreased Muc5AC expression is associated with poor prognosis in gastric cancer. Int J Cancer 134: 114–124. 23801416 10.1002/ijc.28345

[17] Lee HJ, Nam KT, Park HS, Kim MA, Lafleur BJ, et al. (2010) Gene expression profiling of metaplastic lineages identifies CDH17 as a prognostic marker in early stage gastric cancer. Gastroenterology 139: 213–225 e213. 10.1053/j.gastro.2010.04.008 PMC2917327 20398667

[18] Baldus SE, Monig SP, Arkenau V, Hanisch FG, Schneider PM, et al (2002) Correlation of MUC5AC immunoreactivity with histopathological subtypes and prognosis of gastric carcinoma. Ann Surg Oncol 9: 887–893. 12417511 10.1007/BF02557526

[19] Lee HS, Lee HK, Kim HS, Yang HK, Kim WH (2003) Tumour suppressor gene expression correlates with gastric cancer prognosis. J Pathol 200: 39–46. 12692839 10.1002/path.1288

[20] Reis CA, David L, Correa P, Carneiro F, de Bolos C, et al (1999) Intestinal metaplasia of human stomach displays distinct patterns of mucin (MUC1, MUC2, MUC5AC, and MUC6) expression. Cancer Res 59: 1003–1007. 10070955

[21] Young HWJ, Williams OW, Chandra D, Bellinghausen LK, Pérez G, et al (2007) Central Role of Muc5ac Expression in Mucous Metaplasia and Its Regulation by Conserved 5′ Elements. American Journal of Respiratory Cell and Molecular Biology 37: 273–290. 17463395 10.1165/rcmb.2005-0460OC PMC1994232

[22] Jonckheere N, Van Der Sluis M, Velghe A, Buisine M-P, Sutmuller M, et al (2004) Transcriptional activation of the murine Muc5ac mucin gene in epithelial cancer cells by TGF-beta/Smad4 signalling pathway is potentiated by Sp1. Biochem J 377: 797–808. 14570593 10.1042/BJ20030948 PMC1223907

[23] Jonckheere N, Vincent A, Franquet-Ansart H, Witte-Bouma J, Korteland-van Male A, et al (2012) GATA-4/-6 and HNF-1/-4 families of transcription factors control the transcriptional regulation of the murine Muc5ac mucin during stomach development and in epithelial cancer cells. Biochimica et Biophysica Acta (BBA) - Gene Regulatory Mechanisms 1819: 869–876. 22554936 10.1016/j.bbagrm.2012.04.003

[24] Di YP, Zhao J, Harper R (2012) Cigarette Smoke Induces MUC5AC Protein Expression through the Activation of Sp1. Journal of Biological Chemistry 287: 27948–27958. 22700966 10.1074/jbc.M111.334375 PMC3431669

[25] Inaguma S, Kasai K, Ikeda H (2011) GLI1 facilitates the migration and invasion of pancreatic cancer cells through MUC5AC-mediated attenuation of E-cadherin. Oncogene 30: 714–723. 20972463 10.1038/onc.2010.459

[26] Mori Y, Kataoka H, Miura Y, Kawaguchi M, Kubota E, et al (2007) Subcellular localization of ATBF1 regulates MUC5AC transcription in gastric cancer. International Journal of Cancer 121: 241–247. 17330845 10.1002/ijc.22654

[27] Hui C-c, Angers S (2011) Gli Proteins in Development and Disease. Annual Review of Cell and Developmental Biology 27: 513–537. 10.1146/annurev-cellbio-092910-154048 21801010

[28] Yamamichi N, Inada K, Ichinose M, Yamamichi-Nishina M, Mizutani T, et al (2007) Frequent loss of Brm expression in gastric cancer correlates with histologic features and differentiation state. Cancer Res 67: 10727–10735. 18006815 10.1158/0008-5472.CAN-07-2601

[29] Yamamichi N, Yamamichi-Nishina M, Mizutani T, Watanabe H, Minoguchi S, et al (2005) The Brm gene suppressed at the post-transcriptional level in various human cell lines is inducible by transient HDAC inhibitor treatment, which exhibits antioncogenic potential. Oncogene 24: 5471–5481. 16007216 10.1038/sj.onc.1208716

[30] Larkin MA, Blackshields G, Brown NP, Chenna R, McGettigan PA, et al (2007) Clustal W and Clustal X version 2.0. Bioinformatics 23: 2947–2948. 17846036 10.1093/bioinformatics/btm404

[31] Li D, Gallup M, Fan N, Szymkowski DE, Basbaum CB (1998) Cloning of the Amino-terminal and 5′-Flanking Region of the HumanMUC5AC Mucin Gene and Transcriptional Up-regulation by Bacterial Exoproducts. Journal of Biological Chemistry 273: 6812–6820. 9506983 10.1074/jbc.273.12.6812

[32] Kinzler KW, Vogelstein B (1990) The GLI gene encodes a nuclear protein which binds specific sequences in the human genome. Mol Cell Biol 10: 634–642. 2105456 10.1128/mcb.10.2.634-642.1990 PMC360861

[33] Yoon JW, Kita Y, Frank DJ, Majewski RR, Konicek BA, et al (2002) Gene expression profiling leads to identification of GLI1-binding elements in target genes and a role for multiple downstream pathways in GLI1-induced cell transformation. J Biol Chem 277: 5548–5555. 11719506 10.1074/jbc.M105708200

[34] Zhu H, Lo HW (2010) The Human Glioma-Associated Oncogene Homolog 1 (GLI1) Family of Transcription Factors in Gene Regulation and Diseases. Curr Genomics 11: 238–245. 21119888 10.2174/138920210791233108 PMC2930663

[35] Xu XF, Guo CY, Liu J, Yang WJ, Xia YJ, et al (2009) Gli1 maintains cell survival by up-regulating IGFBP6 and Bcl-2 through promoter regions in parallel manner in pancreatic cancer cells. J Carcinog 8: 13. 19736394 10.4103/1477-3163.55429 PMC2746911

[36] Bien-Willner GA, Stankiewicz P, Lupski JR (2007) SOX9cre1, a cis-acting regulatory element located 1.1 Mb upstream of SOX9, mediates its enhancement through the SHH pathway. Hum Mol Genet 16: 1143–1156. 17409199 10.1093/hmg/ddm061

[37] El-Zaatari M, Kao JY, Tessier A, Bai L, Hayes MM, et al (2013) Gli1 deletion prevents Helicobacter-induced gastric metaplasia and expansion of myeloid cell subsets. PLoS One 8: e58935. 23520544 10.1371/journal.pone.0058935 PMC3592845

[38] Wang F, Xu L, Guo C, Ke A, Hu G, et al (2011) Identification of RegIV as a novel GLI1 target gene in human pancreatic cancer. PLoS One 6: e18434. 21494603 10.1371/journal.pone.0018434 PMC3073946

[39] Machado JC, Nogueira AM, Carneiro F, Reis CA, Sobrinho-Simoes M (2000) Gastric carcinoma exhibits distinct types of cell differentiation: an immunohistochemical study of trefoil peptides (TFF1 and TFF2) and mucins (MUC1, MUC2, MUC5AC, and MUC6). J Pathol 190: 437–443. 10699992 10.1002/(SICI)1096-9896(200003)190:4<437::AID-PATH547>3.0.CO;2-1

[40] Ito T, Yamauchi M, Nishina M, Yamamichi N, Mizutani T, et al (2001) Identification of SWI.SNF complex subunit BAF60a as a determinant of the transactivation potential of Fos/Jun dimers. J Biol Chem 276: 2852–2857. 11053448 10.1074/jbc.M009633200

[41] Dallas PB, Cheney IW, Liao DW, Bowrin V, Byam W, et al (1998) p300/CREB binding protein-related protein p270 is a component of mammalian SWI/SNF complexes. Mol Cell Biol 18: 3596–3603. 9584200 10.1128/mcb.18.6.3596 PMC108941

[42] de la Serna IL, Carlson KA, Imbalzano AN (2001) Mammalian SWI/SNF complexes promote MyoD-mediated muscle differentiation. Nat Genet 27: 187–190. 11175787 10.1038/84826

[43] Im H, Grass JA, Johnson KD, Kim SI, Boyer ME, et al (2005) Chromatin domain activation via GATA-1 utilization of a small subset of dispersed GATA motifs within a broad chromosomal region. Proc Natl Acad Sci U S A 102: 17065–17070. 16286657 10.1073/pnas.0506164102 PMC1287986

[44] Benson G (1999) Tandem repeats finder: a program to analyze DNA sequences. Nucleic Acids Research 27: 573–580. 9862982 10.1093/nar/27.2.573 PMC148217

[45] Yamada N, Nishida Y, Yokoyama S, Tsutsumida H, Houjou I, et al (2010) Expression of MUC5AC, an early marker of pancreatobiliary cancer, is regulated by DNA methylation in the distal promoter region in cancer cells. Journal of Hepato-Biliary-Pancreatic Sciences 17: 844–854. 20734208 10.1007/s00534-010-0278-0

